# Power-Efficient Wireless Coverage Using Minimum Number of UAVs

**DOI:** 10.3390/s22010223

**Published:** 2021-12-29

**Authors:** Ahmad Sawalmeh, Noor Shamsiah Othman, Guanxiong Liu, Abdallah Khreishah, Ali Alenezi, Abdulaziz Alanazi

**Affiliations:** 1 Computer Science Department, Northern Border University, Arar 91431, Saudi Arabia; 2 Remote Sensing Unit, Northern Border University, Arar 91431, Saudi Arabia; ali.hamdan@nbu.edu.sa; 3 Department of Electrical and Electronics Engineering, Universiti Tenaga Nasional, Kajang 43000, Selangor, Malaysia; shamsiah@uniten.edu.my; 4 Department of Electrical and Computer Engineering, Newark College of Engineering, New Jersey Institute of Technology, University Heights, Newark, NJ 07102, USA; gl236@njit.edu (G.L.); abdallah@njit.edu (A.K.); 5 Electrical Engineering Department, Northern Border University, Arar 91431, Saudi Arabia; af.alanazi@nbu.edu.sa

**Keywords:** unmanned aerial vehicles (UAVs), efficient 3D placement, *K*-means, Artificial Bees Colony (ABC), Particle Swarm Optimization (PSO), Genetic Algorithm (GA)

## Abstract

Unmanned aerial vehicles (UAVs) can be deployed as backup aerial base stations due to cellular outage either during or post natural disaster. In this paper, an approach involving multi-UAV three-dimensional (3D) deployment with power-efficient planning was proposed with the objective of minimizing the number of UAVs used to provide wireless coverage to all outdoor and indoor users that minimizes the required UAV transmit power and satisfies users’ required data rate. More specifically, the proposed algorithm iteratively invoked a clustering algorithm and an efficient UAV 3D placement algorithm, which aimed for maximum wireless coverage using the minimum number of UAVs while minimizing the required UAV transmit power. Two scenarios where users are uniformly and non-uniformly distributed were considered. The proposed algorithm that employed a Particle Swarm Optimization (PSO)-based clustering algorithm resulted in a lower number of UAVs needed to serve all users compared with that when a *K*-means clustering algorithm was employed. Furthermore, the proposed algorithm that iteratively invoked a PSO-based clustering algorithm and PSO-based efficient UAV 3D placement algorithms reduced the execution time by a factor of ≈1/17 and ≈1/79, respectively, compared to that when the Genetic Algorithm (GA)-based and Artificial Bees Colony (ABC)-based efficient UAV 3D placement algorithms were employed. For the uniform distribution scenario, it was observed that the proposed algorithm required six UAVs to ensure 100% user coverage, whilst the benchmarker algorithm that utilized Circle Packing Theory (CPT) required five UAVs but at the expense of 67% of coverage density.

## 1. Introduction

Unmanned aerial vehicles (UAVs) have become a promising solution in supporting public safety, search and rescue operations and disaster management. In the case of natural disasters such as earthquakes, floods or tsunamis, there are chances that the communication systems’ infrastructures become partially or completely disrupted. Therefore, rapid solutions are necessary to provide wireless coverage in support of rescue operations [1,2].

Generally, the main uses of UAVs as wireless aerial base stations can be classified into different categories based on their roles in different application scenarios [1,3,4]: (i) UAV-aided wireless communication due to cellular outage, where a UAV is used as a backup base station that operates at a much higher altitude to provide ubiquitous coverage when the ground base station completely goes out of service during disastrous situations [5,6,7,8]. (ii) UAV-aided wireless communication during cellular network congestion, where a UAV is used to supplement the existing ground base stations during a massively crowded special event when the cellular network is overloaded [9,10]. (iii) UAV-aided relay communication, where UAVs are used as relay nodes in providing wireless connectivity between two or more distant wireless points when there is no direct communication links or line-of-sight (LoS) due to an obstruction such as hill [11]. (iv) UAV-aided data collection, where UAVs are used to collect data from ground Internet of Things (IoT) devices [12].

In this paper, the deployment of multiple UAVs as backup base stations during natural disasters is considered. More specifically, we consider a multi-UAV-aided system to establish an emergency network that aims to maximize the wireless coverage using the minimum number of UAVs. Here, it is considered that each UAV serves both outdoor and indoor users that are either uniformly or non-uniformly distributed inside the coverage area.

## 2. Related Works

Recently, there has been an extensive amount of research related to the deployment of UAVs as aerial base stations. The optimal location of UAVs is one of the main issues that must be addressed in the case of UAV-aided wireless communication due to cellular outage or during cellular network congestion. The problem of finding the optimal location of a UAV is formulated with various objective functions such as to maximize wireless coverage or network throughput, to maximize user coverage probability and to minimize power consumption.

The authors in [7,8,10,13,14,15,16] studied the case of the deployment of a single UAV as an aerial base station to serve outdoor users with different objective functions. However, these studies utilized the path loss model in [17], where a statistical propagation model was proposed to predict the Air-To-Ground (ATG) pathloss between UAVs in low-altitude platforms (LAPs) and ground nodes. The authors in [8] studied the problem of finding the optimal altitude of a single UAV that aimed to provide maximum wireless coverage with minimum UAV transmit power. The authors also studied the case of two UAVs that provided maximum coverage in the presence and absence of interference. The study in [13] proposed a model to find an efficient UAV 3D position that aimed to maximize the network throughput. In [14], the authors proposed an optimal 3D placement algorithm for the deployment of a single UAV base station that aimed to maximize the total number of covered users by imposing the minimum required UAV transmit power constraint. The authors in [16] studied an on-demand UAV placement problem for arbitrarily distributed users. The problem was formulated with the objective of maximizing the covered users for different user densities with guaranteed data rates.

On the other hand, the authors in [7] proposed a model to find an efficient 3D placement of a single UAV that served indoor users by utilizing the outdoor to indoor pathloss model of [18] certified by the International Telecommunication Union (ITU). The objective was to minimize the total required UAV transmit power such that all indoor users were covered. Furthermore, the authors in [10] studied the problem of finding the optimal altitude of a single UAV that served outdoor and indoor users simultaneously and aimed to minimize the total required UAV transmit power and maximize the coverage area.

The work presented so far is related to the deployment of a single UAV that serves outdoor and/or indoor users within small coverage areas. Thus, in the case of users within a large coverage area, the deployment of multiple UAVs is required.

The studies in [19,20,21] considered the cellular networking scenario where UAVs were deployed to support the connectivity of existing terrestrial wireless networks. The study in [19] proposed an approach to deploy multi-UAVs coexisting with a ground base station to provide wireless coverage for users in a crowded region. In this work, the efficient number and the 3D placement of UAVs were found in such that the traffic demands were satisfied, with arbitrary user distribution, and the system sum-rate was maximized. In addition, this work considered the problem of co-channel interference. In [20], the authors proposed a coordination and cooperation model where a UAV was used to assist the terrestrial cellular network. Specifically, the authors developed a cooperative UAV clustering approach to offload ground mobile terminals from ground cellular base stations to cooperative UAV clusters. Meanwhile, the work in [21] addressed the issue in the backhaul links between drones and ground base stations. More specifically, the authors in [21] proposed a 3D UAV placement and trajectory model. Bezier curves were utilized to achieve the best coverage for clusters of ground terminals.

Another main issue to be considered in the deployment of multi-UAVs is finding the number of UAVs to be deployed. The studies in [9,22,23,24] considered the deployment of multi-UAVs equipped with a directional antenna that has a circular coverage pattern. The authors in [25] proposed an approach using a heuristic algorithm to find the minimum number of UAVs along with their placements to provide coverage for outdoor users. The coverage area was divided into equal regions and the users were distributed uniformly with different densities. In [23], the authors proposed an optimal 3D deployment strategy of multi-UAVs that used Circle Packing Theory (CPT). In this work, the optimal 3D location of the UAVs were determined with the aim to maximize the circular coverage area. A similar approach was presented in [9], where the authors proposed to utilize CPT to find the number of UAVs with the aim to maximize the coverage density while ensuring the coverage area of each UAV did not overlap. However, the authors considered three different shapes of wireless coverage area, namely square, rectangular and circular regions. Here, the CPT was utilized in tandem with the 3D efficient placement algorithm.

Another interesting approach is the deployment of multi-UAVs by employing a clustering algorithm [26,27,28,29,30]. The authors in [26] proposed an algorithm to position UAVs in order to complement the macrocell infrastructure. The *K*-means clustering algorithm was used to partition user equipments (UEs) into *K* subsets, and a decision was made on which subsets were to be serviced by UAVs. The centroid of the subset was set as the 2D location of the UAV which served the UEs within the subset to offload from the macrocells. In [27], the authors studied a similar approach as the work in [26]; however, the positioning of multi-UAVs and the association of the UEs were jointly optimized and aimed to maximize the number of UEs and satisfy the UE’s experienced data rate (represented via bandwidth allocation). Two jointly optimized algorithms based on Particle Swarm Optimization (PSO) and the Genetic Algorithm (GA) were proposed. Both proposed algorithms improved the UE’s satisfaction with the provided data rates when compared with the proposed algorithm that invoked a *K*-means algorithm.

However, both works in [26,27] considered the 2D placement of UAVs that served the associated UEs.

The authors in [28] studied the 3D placement of a single UAV as an aerial base station to serve indoor users alone inside a high-rise building. The problem was formulated with the aim to minimize the number of UAVs required to serve all indoor users. This work considered the indoor users that were distributed uniformly with the pathloss model of [18]. The proposed solution used a *K*-means clustering algorithm to partition the indoor users into an initial *k* cluster and then exploited PSO to find an efficient 3D position of UAV subjected to the constraint that the total transmit power was smaller than the threshold value. A single UAV was assigned to each cluster. The number of clusters was increased if the total transmit power constraint was not met. This process was performed iteratively until the total transmit power constraint was met.

In [29], the authors proposed a UAV-aided emergency rescue network where each UAV served as a wireless base station. The problem was formulated with the objective to minimize the number of UAVs to cover all points of interest (POIs), which were locations with a large number of people, for example, schools, hospitals or parks. This work considered the location of POIs that were distributed uniformly and were located outdoors and indoors, with the pathloss model of [17,18], respectively. The authors proposed a similar solution as the work in [28], where an iterative process was performed to partition the POIs into *k* clusters using a *K*-means clustering algorithm and finding an efficient 3D position of the UAV. However, this work exploited a Genetic Algorithm (GA) to find the efficient 3D position of a UAV in providing wireless coverage to POIs that were located outdoors and indoors.

In [30], the authors proposed a multi-UAV deployment strategy for resource allocation in a UAV-enabled mobile edge computing (MEC) network. This work aimed to minimize the sum power consumption of UEs and UAVs that included both communication-related power and mechanical power with latency and coverage constraints. The sum power minimization problem was decomposed into three subproblems on user association, computation capacity allocation and location planning. The proposed algorithm iteratively invoked the algorithm to solve the three subproblems. In this work, the Fuzzy C-means clustering algorithm was proposed to solve the joint user association and location planning subproblems. The proposed algorithm could efficiently reduce the sum power consumption after three iterations, indicating the reduction in the number of UAVs used.

Meanwhile, in [24], the authors studied the efficient placement of multi-UAVs in such that the user coverage probability was maximized and inter-cell interference (ICI) was avoided. More specifically, the authors proposed a non-overlapped circle placement method to find the optimal UAV placement in such that the circle covered the maximum number of users by considering the required total transmit power. In this work, the outdoor users were randomly distributed using spatial points processes (SPPs) and were partitioned into *K* clusters using a *K*-means algorithm; subsequently, the optimal UAV placement was determined using the proposed algorithm in such that the user coverage probability was maximized while the total transmit power was minimized. The user coverage probability and the power efficiency were further improved using the proposed iterative algorithms.

However, the clustering algorithm used in [24,26,28,29] exploited a *K*-means algorithm to partition users into *K* clusters.

A *K*-means algorithm is relatively a simple algorithm and is easy to implement, which makes it the most popular algorithm that is used in several fields. However, a *K*-means algorithm is very sensitive to the initial cluster centers. More specifically, in *K*-means algorithms, the initial cluster centers are chosen randomly; this may cause three possible problems: (i) The final clustering results are not unique. For different runs of the same input data, the algorithm produces different clusters [31]. (ii) The initial cluster center has an influence on the number of iterations. The number of iterations required for the algorithm to converge is high if the chosen initial cluster center is located far away from the final cluster center [31]. (iii) The algorithm may converge in local minimum instead of global minimum, which results in a sub-optimal solution [32,33]. Furthermore, the *K*-means algorithm is sensitive to outliers and noisy data. The outliers have an influence on the clustering of data points that results in some data points being clustered incorrectly [32,33]. Moreover, the algorithm produces clusters that are not symmetric, where the clusters have unequal sizes and densities (unequal numbers of data points in each cluster) [34].

Various methods have been proposed in the literature to mitigate the drawbacks of *K*-means algorithms. The authors in [35,36] proposed a GA-based clustering algorithm as a solution to the problem that a *K*-means algorithm may get stuck at sub-optimal solutions due to a poor randomly chosen initial cluster center. Meanwhile, the application of PSO in data clustering was discussed in [32]. It has been demonstrated that both GA and PSO algorithms overcome the problem associated with the *K*-means algorithm that tends to trap in local optima [32,35,36].

Meanwhile, the work in [28,29] considered the location of users/POIs that were distributed uniformly. In [26], three set of UEs were distributed with three different distributions, namely Poisson, random and uniform distribution, whilst in [24], the authors considered the users to be distributed using SPP.

With regard to the energy efficiency approach, the authors in [37] proposed an iterative algorithm to solve the energy efficiency problem with the objective to maximize the ratio of the ergodic total data size to the total energy consumption that included both transmit power and propulsion power to hover. The problem was decomposed into two subproblems on finding optimal coordinated power allocation and finding optimal hovering time scheduling. The proposed iterative algorithm resulted in higher energy efficiency as the number of UAVs increased, due to higher diversity gain and flexibility in the coordination of aerial small cells. This observation was obtained because more UAVs resulted in less hovering time for a fixed total transmit energy. Thus, the transmit power for each UAV was higher; hence, the total data size increased. In [38], it was stated that the energy consumption of a UAV refers to the conventional communications-related energy consumption and propulsion energy consumption. However, this work focuses on the communications-related energy consumption alone.

In this paper, we propose a power-efficient algorithm that maximizes the coverage area using the minimum number of UAVs that aims to minimize the required UAV transmit power while satisfying the required users’ data rate. The proposed algorithm considers the deployment of multi-UAVs that serve outdoor and indoor users by iteratively invoking a clustering algorithm and an efficient 3D UAV placement algorithm.

More specifically, we study the proposed algorithm that invokes three variants of clustering algorithms that are developed based on a *K*-means algorithm, PSO and GA for partitioning both outdoor and indoor users into *k* clusters. Here, we define user partitioning as dividing coverage area into *k* small subareas. In this study, we consider two scenarios where users are distributed uniformly and non-uniformly using beta function. Subsequently, the proposed algorithm invokes three variants of efficient 3D UAV placement algorithms that are developed based on PSO, GA and Artificial Bees Colony (ABC).

### Paper Contribution

The contributions of this paper are summarized as follows:

- *K*-means and meta-heuristic clustering algorithms, based on PSO and GA, respectively, are utilized for partitioning outdoor and indoor users into clusters which correspond with partitioning the disaster-affected area with the condition that the UAV transmit power for each cluster is minimized. The employment of the meta-heuristic algorithm is superior in comparison with the *K*-means based clustering algorithm in terms of the cluster quality, where the resulting clusters are symmetrical.
- The efficient UAV 3D placement algorithm based on the ABC algorithm is proposed, with the aim to minimize the required UAV transmit power while satisfying the data rate requirement. The employment of each of the three variants of the efficient UAV 3D placement algorithm, namely PSO-based, GA-based and ABC-based algorithms, are evaluated in terms of the computational complexity which is manifested in terms of its execution time taken.
- A power-efficient algorithm is proposed that iteratively invokes a clustering algorithm and an efficient UAV 3D placement algorithm that aims to minimize the number of UAVs to serve outdoor and indoor users simultaneously, while minimizing each UAV transmit power. The proposed algorithm attained 100% coverage density, which corresponds with providing wireless coverage to all users that are uniformly and non-uniformly distributed using the minimum number of UAVs. Furthermore, the proposed algorithm that invokes a PSO-based clustering algorithm resulted in a lower number of required UAVs that served all outdoor and indoor users compared to that when the *K*-means clustering algorithm was employed.

Section 3 introduces the system model. Section 4 presents the problem formulation and the proposed algorithm to find the minimum number of UAVs that aims to maximize wireless coverage by imposing the constraint to minimize the required UAV transmit power. This is followed by the clustering algorithms that are invoked to partition users by the proposed algorithm in Section 5. Then, Section 6 presents three variants of efficient UAV 3D placement algorithms based on PSO, GA and ABC algorithms. Section 7 quantifies the comparison performance of the three clustering algorithms variants, as well as the performance of the proposed power-efficient algorithm that iteratively invokes a clustering algorithm and an efficient UAV 3D placement algorithm for the case when users are distributed uniformly and non-uniformly. Finally, Section 8 concludes the paper.

## 3. System Model

**Figure 1:**
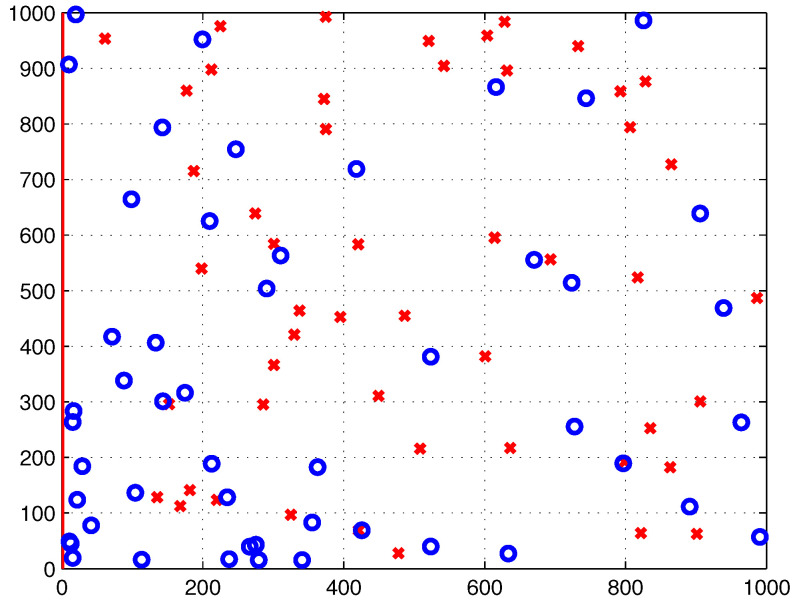
The distribution of outdoor and indoor users inside the disaster-affected area, S.

Consider an area where a natural disaster occurs, denoted as S. Figure 1 illustrates the affected area, S, with minimum and maximum points of ($x_{min}$, $y_{min}$) and ($x_{max}$, $y_{max}$), respectively.

This work aims for an power-efficient deployment strategy of multi-UAVs that simultaneously serves all outdoor and indoor users within the disaster-affected area, S. More specifically, in this work, three different clustering algorithms are invoked for use in partitioning the users which corresponds to partitioning the coverage area, S, into *n* subareas. One UAV is assigned to serve each subarea, $S_{n}$. Moreover, this work considers two scenarios of user distribution inside the coverage area, namely uniform distribution and non-uniform distribution using beta random distribution, denoted as function f(x,y). In this work, the MATLAB beta random generation function *betarnd (A, B, m, n)* is used to generate an *m*-by-*n* array. This array contains random numbers from the beta distribution with parameters *A* and *B*, with A=1, B=1, m=1 and *n* = number of users inside coverage area. Figure 1 illustrates the distribution of outdoor and indoor users within the disaster-affected area, S, which is represented as blue circles and red crosses, respectively.

### Path Loss Models

This section presents the Air-To-Ground (ATG) [17] and Outdoor-to-Indoor [18] path loss models that are utilized when considering the deployment of UAVs to serve outdoor and indoor users, respectively.

For the ATG channel modeling in an urban environment, both the line-of-sight (*LOS*) links and the non-line-of-sight (*NLOS*) links between the UAV and the ground users are considered. The propagation conditions in the probabilistic LAP model [17] have been extensively used for coverage analysis and for finding the optimal UAV position [8,23]. The probability of *LOS* and *NLOS* can be calculated based on the relative locations between UAVs and ground outdoor users. The average path loss, $PL_{out}\left(dB\right)$, of the ATG channel model can be formulated as:

(1) $$\begin{array}{c} PL_{out}\left(dB\right)=P_{LOS}\times L_{LOS}+P_{NLOS}\times L_{NLOS,} \end{array}$$

where the path loss for the *LOS* link, $L_{LOS}$, and the *NLOS* link, $L_{NLOS}$, as well as the probability of *LOS*, $P_{LOS}$, and *NLOS* link, $P_{NLOS}$, are given as [17]:  

(2) $$\begin{array}{c} L_{LOS}=20log\left(\frac{4\pi f_{c}d}{c}\right)+\eta_{LOS},\\ L_{NLOS}=20log\left(\frac{4\pi f_{c}d}{c}\right)+\eta_{NLOS},\\ P_{LOS}=\frac{1}{1+a\cdot exp(-b\left[\left(\frac{180}{\pi }\right)\theta -a\right])},\\ P_{NLOS}=1-P_{LOS} \end{array}$$

where *c* is light speed, $f_{c}$ is the carrier frequency and $\eta_{LOS}$, $\eta_{NLOS}$ are additional losses depending on the environment. Meanwhile, *a* and *b* are constants that depend on the environment. θ is the elevation angle between the UAV and ground user, *h* is the altitude of the UAV and *d* is the distance between the UAV and the ground user.

Meanwhile, the Outdoor-to-Indoor path loss model of [18] is utilized when considering wireless coverage for indoor users, which is given as:

(3) $$\begin{array}{c} PL_{in}\left(dB\right)=PL_{PFS}+PL_{PB}+PL_{IN},\\ PL_{PFS}=20log\left(d_{3D}\right)+20log\left(f_{c}\right)+a_{1},\\ PL_{PB}=a_{2}+a_{3}{\left(1-cos\theta \right)}^{2},\\ PL_{IN}=a_{4}\cdot d_{2D_{in}} \end{array}$$

where $PL_{PFS}$ is the path loss in the free space, $PL_{PB}$ is the penetration loss of the building and $PL_{IN}$ is the indoor path loss, $d_{3D}$ is the Euclidean distance between the UAV and the indoor user *i*, $f_{c}$ is the carrier frequency, θ is the elevation angle, $d_{2D_{in}}$ is the 2D indoor distance between the UAV and indoor user *i* and $a_{1},a_{2},a_{3}$ and $a_{4}$ are environmental constant values.

## 4. Problem Formulation

In this section, the deployment of multi-UAVs to provide wireless coverage for outdoor and indoor users using the minimum number of UAVs during a natural disaster is formulated as an optimization problem.

Consider a disaster-affected area, S. Let the dimension of the area be denoted by $\left[0,x_{max}\right]$ × $\left[0,y_{max}\right]$. A set of $M_{out}$ outdoor users and $M_{in}$ of indoor users are distributed inside the area. The 2D user location is represented with their location vector P = ($p^{x},p^{y}$). The problem of finding the minimum number of UAVs that provide wireless coverage for all outdoor and indoor users inside S and their efficient 3D placements can be formulated as follows:   

(4a) $$\begin{array}{c} \underset{x_{u},y_{u},z_{u}}{\mathrm{minimize}}\;\;\;K \end{array}$$

(4b) $$\begin{array}{c} \mathrm{subject}\;\mathrm{to}\;\;\sum_{o=1}^{M_{out}^{k}}P_{out_{o}}+\sum_{i=1}^{M_{in}^{k}}P_{in_{i}}\leq P_{UAV_{max}}, \end{array}$$

(4c) $$\begin{array}{c} \sum_{k=1}^{K}\left(M_{out}^{k}+M_{in}^{k}\right)=(N_{out}+N_{in}), \end{array}$$

(4d) $$\begin{array}{c} P_{j}\cap P_{\bar{j}}=⌀\;\;\;\;\;\forall j\;\neq \bar{j}, \end{array}$$

(4e) $$\begin{array}{c} B_{k}=\frac{B}{K}, \end{array}$$

(4f) $$\begin{array}{c} x_{min}\leq x_{i_{UAV_{k}}}\leq x_{max}, \end{array}$$

(4g) $$\begin{array}{c} y_{min}\leq y_{i_{UAV_{k}}}\leq y_{max}, \end{array}$$

(4h) $$\begin{array}{c} z_{min}\leq z_{i_{UAV_{k}}}\leq z_{max} \end{array}$$

where *K* is defined as the total number of UAVs that are deployed to serve all outdoor and indoor users inside S and ($x_{u},y_{u},z_{u}$) is the UAV 3D placement that minimizes its transmit power.

The first constraint of Equation (4b) guarantees that the total power required to cover all users is less than the threshold power of UAV $P_{UAV_{max}}$, where the total required power for outdoor and indoor users that satisfies the minimum data rate $R_{b}$ can be represented as follows:

(5) $$\begin{array}{c} P_{out_{tot}}=\sum_{o=1}^{M_{out}^{k}}\left[\left(2^{\frac{R_{b}.M_{out}^{k}}{B}}-1\right)\times N_{p}\times PL_{out_{o}}\right]\\ P_{in_{tot}}\;=\;\sum_{i=1}^{M_{in}^{k}}\left[\left(2^{\frac{R_{b}.M_{in}^{k}}{B}}-1\right)\times N_{p}\times PL_{in_{i}}\right] \end{array}$$

The constraint of Equations (4c) and (4d) ensure that there is no overlapping between all users that are served by $k^{th}$ UAV and other users that are not served by the $k^{th}$ UAV. In Equation (4c), $M_{out}^{k_{th}}$ and $M_{in}^{k_{th}}$ denote the the outdoor and indoor users that are served by $k^{th}$ UAV, whilst $\left(N_{out}+N_{in}\right)$ defines the total number of users inside S. In Equation (4d), $P_{j}$ denotes the 2D location vector of each user that is served by the $k^{th}$ UAV, whilst $P_{\bar{j}}$ denotes the 2D location vector of each user that is not served by the $k^{th}$ UAV.

In the constraint of Equation (4e), the total available bandwidth, *B*, is divided evenly between UAVs, where $B_{k}$ denotes the bandwidth that is allocated for each UAV. The constraint of Equations (4f)–(4h) ensure that all UAVs are located within the range of minimum and maximum values inside the coverage area.

In this paper, we assume that the interference is implicitly modeled as noise. In the system model, Frequency Division Multiple Access (FDMA) is used. It is assumed that each UAV allocates equal channel bandwidth to ground users, and in order to avoid interference, each channel is assigned to one user.

**Figure 2:**
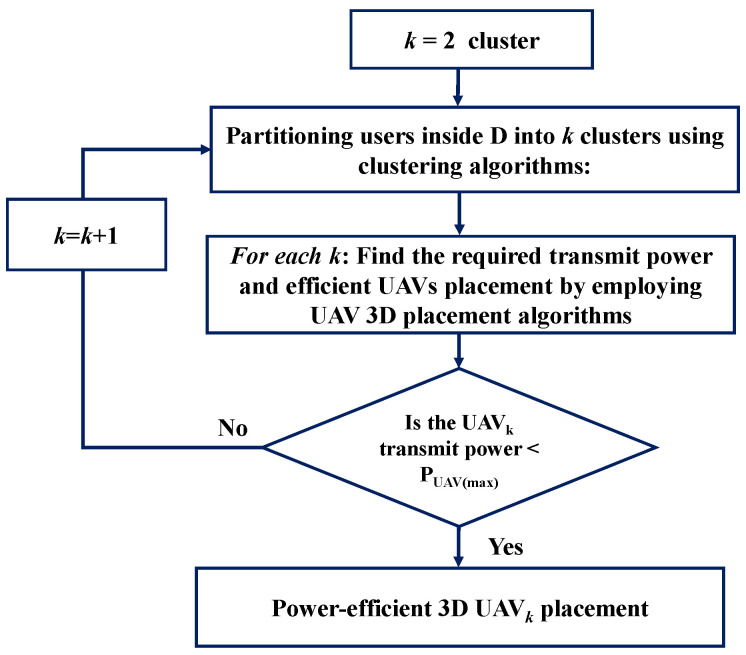
Flowchart of the proposed heuristic approach.

Clearly, finding the minimum number of UAVs that serve all users inside S and their efficient 3D placements such that the total transmit power of each UAV is minimized makes the problem very complicated. Therefore, this optimization problem is an NP-hard problem [39] and can be solved using meta-heuristic algorithms. Algorithm 1 shows the pseudocode of the proposed algorithm to solve the formulated problem similar to the approach in [28,29]. More specifically, the proposed approach to solve the formulated problem can be performed in the following stages, as illustrated in Figure 2:

1. Initially, the number of UAVs that are used to serve all users inside the coverage area S is set as k=2.
2. Then, the proposed clustering algorithm is invoked to partition the users into *k* clusters. The proposed three variants of clustering algorithm based on *K*-means algorithm, PSO and GA are presented in Section 5.
3. The UAV 3D placement for each *k* cluster is determined by invoking the proposed efficient UAV 3D placement algorithm. Section 6 presents the three variants of the UAV efficient 3D placement algorithm.
4. The required total transmit power to provide wireless coverage to all users inside S is determined using Equation (4b). If the UAV transmit power ≥ $P_{UAV_{max}}$, then the value of *k* is increased by 1. In other words, the number of clusters of users inside S is increased by 1.
5. An iterative process of stage (2) to (4) is performed until the constraint of Equation (4b) is met. In this work, we use $P_{UAV_{max}}=1\;\mathrm{watt}$.

The computational complexity of the proposed heuristic approach is the summation of the complexity of the clustering algorithm and the complexity of the efficient UAV 3D placement algorithm.
**Algorithm 1:** Proposed heuristic approach.1. STEP 1: **Input**: Coverage region S; p = ($p^{x},p^{y}$) set of users location; *k* clusters.2. STEP 2: **Repeat:**3. Partitioning users inside S into *k* clusters using *K*-means, PSO and GAalgorithms. For each cluster an efficient UAV placement is found usingPSO and GA Algorithms such that:4.                $P_{out_{tot}}+P_{in_{tot}}$≤ $P_{UAV_{max}}$, where5.                $P_{out_{tot}}=\sum_{o=1}^{M_{out}}\left[\left(2^{\frac{r\cdot M_{out}}{B}}-1\right)\times N_{p}\times PL_{out_{o}}\right]$6.                $P_{in_{tot}}\;=\;\sum_{i=1}^{Min}\left[\left(2^{\frac{r\cdot M_{in}}{B}}-1\right)\times N_{P}\times PL_{in_{i}}\right]$7. STEP 3: **If** UAV transmission power ≥ $P_{UAV_{max}}$:8.                Increase the number of clusters by 1.9.                Go to Step 2.10. STEP 3: **else**11.                Output = UAV${}_{k}$ 3D placement12. STEP 4: **end****Output:** Set of UAVs 3D placement

For the sake of benchmarking, the multi-UAV deployment approach that utilizes CPT is used for comparison with the proposed power-efficient algorithm. The benchmarker multi-UAV deployment is only considered for the scenario where all users are uniformly distributed.

More specifically, the performance of the proposed power-efficient algorithm is compared with the efficient 3D placement of multi-UAVs that utilizes CPT to serve users in a square shape coverage region of [9]. The benchmarker multi-UAVs deployment approach invokes CPT to find the optimal packing of $n_{c}$ non-overlapped and identical circles into a unit square. Then, the altitude of each UAV is found using the algorithms presented in Section 6.

The problem of packing $n_{c}$ identical circles inside a unit square with the objective to maximize the radius $r_{d}$ of the packed circles such that the coverage density is maximized can be formulated as [9]:   

(6a) $$\begin{array}{c} \underset{x_{i},y_{i}}{\mathrm{minimize}}\;\;\;r_{d} \end{array}$$

(6b) $$\begin{array}{c} \mathrm{subject}\;\mathrm{to}\;\;r_{d}\leq x_{i}\leq 1-r_{d},\;\;\;\;\;\;\;\;\forall i\;\epsilon \;I=\left(1,\ldots ,n_{c}\right), \end{array}$$

(6c) $$\begin{array}{c} r_{d}\leq y_{i}\leq 1-r_{d},\;\;\;\;\;\;\;\;\forall i\;\epsilon \;I=\left(1,\ldots ,n_{c}\right), \end{array}$$

(6d) $$\begin{array}{c} \sqrt{{\left(x_{i}-x_{j}\right)}^{2}+{\left(y_{i}-y_{j}\right)}^{2}}\geq 2r_{d},\;\;\;\;\;\;\;\;\forall \;i\neq j, \end{array}$$

(6e) $$\begin{array}{c} \left(x_{i},y_{i}\right)\;\epsilon \;\left[0,1\right]\;\;\;\;\;\;\;\;\forall i\in I=\left(1,\ldots ,n_{c},\right) \end{array}$$

where ($x_{i},y_{i}$) is the center coordinates of the *i*th circle, $\sqrt{{\left(x_{i}-x_{j}\right)}^{2}+{\left(y_{i}-y_{j}\right)}^{2}}$ is the Euclidean distance between the centers of circles *i* and *j* and $r_{d}$ is the radius of each packed circle. All packed circles are guaranteed to lie inside the square and there is no overlapping between packed circles by imposing the constraint of Equations (6b)–(6d).

The density $d_{n}$ of packing $n_{c}$ identical and non-overlapped circles with radii $r_{d}$ inside a unit square is defined as the ratio of the packed $n_{c}$ circles area to the square area as in the following Equation [9]:

(7) $$\begin{array}{c} d_{n}=n_{c}r_{d}^{2}\pi \end{array}$$

## 5. Clustering Approaches

This section presents two different clustering approaches which may be referred to as iterative distance-based clustering and meta-heuristic clustering algorithms. The objective of the clustering problem formulation is to minimize the Euclidean distance between each user and the centroid, as presented in Section 5.1. Section 5.2 and Section 5.3 present the iterative distance-based clustering and meta-heuristic clustering algorithms, respectively.

One of the most well-known clustering algorithms that falls in the iterative distance-based clustering algorithm category is *K*-means. In [28,29], a *K*-means-based clustering algorithm was employed to partition users and POIs in the case of the deployment of multi-UAVs as aerial base sations. As discussed in Section 2, the clustering problem of the *K*-means algorithm can also be mitigated by utilizing meta-heuristic algorithms.

Thus, this paper extends the clustering approach based on the meta-heuristic clustering algorithm by invoking PSO, as presented in Section 5.3.2. The performances of the three clustering algorithms are compared in terms of algorithm robustness to the outliers and computational complexity, as presented in Section 7.

### 5.1. Mathematical Formulation of the Clustering Problem

The clustering algorithm involves the process of gathering similar data points into the same cluster. Therefore, a similarity metric between two points must be defined. Euclidean distance is the most common distant metric used in clustering algorithms that aims to minimize the total variations within each cluster [40,41]. Thus, the clustering problem can be formulated with the objective to minimize the sum of squared Euclidean distances between each data point with the cluster center, as follows:

(8a) $$\begin{array}{c} \mathrm{minimize}\;F=\sum_{k=1}^{K}\sum_{n=1}^{N}W_{nk}∥x_{n}-c_{k}∥ \end{array}$$

(8b) $$\begin{array}{c} \mathrm{subject}\;\mathrm{to}\;\;\sum_{k=1}^{K}W_{nk}=1\;\;\;\;\;\;\;\;\;\;\;\;\;\;\;\;\;\;\;n=1,\ldots ,N, \end{array}$$

(8c) $$\begin{array}{c} \sum_{n=1}^{N}W_{nk}\geq 1\;\;\;\;\;\;\;\;\;\;\;\;\;\;\;\;\;\;\;k=1,\ldots ,K, \end{array}$$

(8d) $$\begin{array}{c} k\in [1,\ldots ,K], \end{array}$$

(8e) $$\begin{array}{c} n\in [1,\ldots ,N] \end{array}$$

where $x_{n}$ is the *n*th value of the data set ∈ N and the center of the cluster *k*, and $c_{k}$ is the average value of all points in cluster *k* which can be determined using Equation (9).

(9) $$\begin{array}{c} c_{k}=\frac{\sum_{i=1}^{N}W_{nk}x_{n}}{\sum_{i=1}^{N}W_{nk}} \end{array}$$

where $W_{nk}$ is a decision weight ∈ $\left\{0,1\right\}$, $W_{nk}$ = 1 if the point *n* belongs to cluster *k* and otherwise if the *n* point does not belong to the cluster *k*.

### 5.2. Iterative Distance-Based Clustering (K-Means)

A *K*-means algorithm is a partitioning clustering algorithm used to group data or objects into clusters [31] which was developed by J. B. Mac Queen in 1967 [42]. A *K*-means algorithm starts by randomly selecting *k* initial means as the cluster centers, referred to as centroids. Then, this algorithm calculates the Euclidean distance from each data point to these centroids, and a cluster is formed based on the shortest Euclidean distance from the data point to the cluster centroid. Once the data points are grouped into clusters, the centroid is replaced by a new mean value that is calculated based on the mean of the points that belong to the cluster. These tasks are performed for several iterations until the algorithm converges and produces *K* final means [31,43]. However, in this work, the number of clusters are set using the proposed heuristic algorithm of Algorithm 1, as described in Section 4. The *K*-means algorithm is described by the pseudocode of Algorithm 2.
**Algorithm 2:** The *K*-means clustering algorithm.**Result**: A set of *K* clusters**Input;***k*: Number of desired clustersData set $D=\left\{d_{i}|i=1,\ldots ,n\right\}$; *n* set of data points.$c_{k}$: set of centers *k* = 1, …, *K*.$u_{k}$: cluster position that minimizes the distance from the data points to the cluster *k* = 1, …, *K***Initialization;**$c_{i}$ = random(num): Arbitrarily choose *k* items from D as initial centroids;**Repeat for** ∀j **= 1:**n      Assign *i* to Cluster $c_{k}$ according to the minimum distance from $c_{k}$ center      $u_{i}$ = $\frac{1}{c_{i}}$$\sum_{j\in c_{k}}X_{j},\forall i$: Calculate new centers      $E=\sum_{k=1}^{K}\sum_{i\in c_{k}}d\left(x_{i}-u_{k}\right)=\sum_{k=1}^{K}\sum_{i\in c_{k}}\left|\right|x_{i}-u_{k}{\left|\right|}^{2}$**Until**: *E* does not change

- *K*-means Complexity

The computational complexity of a *K*-means algorithm refers to the total number of Euclidean distance computations. More specifically, in each iteration, for a data set $D=\left\{d_{i}|i=1,\ldots ,n\right\}$, where *n* is the total number of data points and *k* is the number of clusters, the computation complexity is O(nk). Thus, for $N_{it}$ number of iterations, the computational complexity is $O(nkN_{it})$.

### 5.3. Meta-Heuristic Clustering Algorithms

Several meta-heuristic-based clustering algorithms have emerged with nature-inspired designs, namely GA and PSO [33,44]. As discussed earlier, several works on the deployment of multi-UAVs as an aerial base station have employed *K*-means clustering algorithms to partition users. Thus, in this work, we study the deployment of multi-UAVs by invoking the meta-heuristic-based clustering algorithm that provides wireless coverage for both outdoor and indoor users.

#### 5.3.1. Genetic Algorithm (GA)

A Genetic Algorithm (GA) is an optimization technique based on the principles of evolution and natural genetics, which can be used to solve NP-complete global optimization problems. It was demonstrated that the GA-based clustering algorithm provided superior performance in solving clustering problems [35,36].

In the initialization stage, an initial population of *k* number of individuals is created, which are referred to as chromosomes. These individuals represent the legitimate solutions of the given optimization problem. In the case of the clustering problem presented in Section 5.1, these individuals represent the cluster centers which initially are chosen randomly. Each legitimate solution will be evaluated by its fitness, which is linked to the objective function of the clustering problem. Then, the termination criterion will be examined. In this work, the number of iterations, $N_{it}$, refers to the number of generations, which is used to be the termination criterion. If the termination criterion is not met, the highest-fitness individuals are selected to generate the next generation. In this work, the fitness proportional selection is used. Furthermore, the selected individuals, which are referred to as parents, will undergo genetically inspired operators, namely crossover and mutation. Thus, new individuals are generated with improved performance.

The fitness of the new set of legitimate solutions or individuals will be evaluated and the termination criterion will be re-examined. The three GA operators, namely selection of the fittest, crossover and mutation will be repeated during each iterative procedure until the termination criterion is met. After this stage, the algorithm produces a set of the best individuals with the highest fitness, which are the solution to the clustering problem, namely the cluster centers [35]. Algorithm 3 presents the pseudo code for the GA-based clustering algorithm.
**Algorithm 3: **Clustering using Genetic Algorithm.**Result:** A set of *K* Clusters**Input;***k*: Number of desired clustersData set $D=\left\{d_{i}|i=1,\ldots ,n\right\}$; *n* set of data points.**Initialization**: Population p(t) = random(pop); Initialized the population randomly;**Repeat:**       **Fitness computation (ft)**: Compute fitness for population p(t)
clusters are formed by assigning each point in D to one of clusters $k_{j}$ withcenter $c_{j}$ such that:     $\left|\right|x_{i}-c_{j}\left||<|\right|x_{i}-c_{p}\left|\right|$,   ∀p=1,2,…,kFor each cluster the new center is $c^{*}$       $C_{i}^{*}$ = $\frac{1}{n_{i}}$$\sum_{x_{j}\in c_{k}}x_{j},\forall i=1,2,\ldots ,k$The new $C_{i}^{*}$ is replaced by the old center $c_{i}$Select parents using proportional selection for the next Generation (G)Apply the Crossover (C) operator for p(t)Apply the Mutation (M) operator for p(t)

- GA Complexity

The computational complexity of the GA-based clustering algorithm relies on the total number of Euclidean distance computation, as well as the three GA operators, namely selection of the fittest, crossover and mutation. In the selection stage, the best individual will be selected using the fitness proportional selection to be the parents for the new generation of *k* individuals using the crossover and mutation operators. The fitness proportional selection or roulette wheel selection has the computational complexity of O(log(k)). Prior to the selection stage, the Euclidean distance of each data point from each of *k* cluster center, $c_{k}$, will be computed. Hence, it takes O(nk) computations. Thus, for a $N_{it}$ number of iterations, the computational complexity of the GA-based clustering algorithm is $O(N_{it}\cdot nk\log\left(k\right))$.

#### 5.3.2. Particle Swarm Optimization (PSO)

In this work, the new clustering algorithm that invokes Particle Swarm Optimization (PSO) is introduced. The PSO is a population-based evolutionary algorithm that was developed by Kennedy and Eberhart in 1995 [45]. This robust algorithm simulates animals’ social behavior and movement of animal swarms such as schools of fish or flocks of birds, and it is able to solve complex optimization problems.

In PSO, each swarm member is referred to as a particle, where each particle is the candidate solution to the optimization problem. During the movement of the particle swarm, the members of the swarm interact and influence each others’ state. More specifically, each member of the swarm moves cooperatively, which forms the speed and direction of the whole swarm in finding the optimal solution. In the PSO algorithm, the speed and direction are referred to as the velocity and position of each particle which are updated according to its own experience and also that of a neighboring particle. Thus, this algorithm combines both the local and global search in finding the optimal solution.

At the beginning of the PSO-based clustering algorithm for the problem formulated in Section 5.1, the positions of *k* particles are initialized randomly and the velocities of the *k* particles are set to 0. Here, each particle is referred to as the cluster center. Then, the fitness of each particle in the swarm is evaluated based on the objective function of the clustering problem.

For every iteration, each particle compares its fitness with its previous best fitness; the highest fitness is set as the personal best, also known as local best, Lbest value. Then, the best fitness, Lbest, of each particle is also compared with the Lbest of other particles in the swarm, and the swarm global best is updated with the greatest fitness, which is known as global best, GBest [46].

Subsequently, the velocity of each particle is modified towards its Lbest and GBest using Equation (10), and its new position is calculated using Equation (11). The *i*th particle in the swarm changes its velocity and position according to the following equations:

(10) $$\begin{array}{cc} Velocity_{i}\left(t+1\right)= & \;W*Velocity_{i}\left(t\right)+r1*c1*(Lbest_{i}\left(t\right)\\ \; & -Position_{i}\left(t\right))+r2*c2*\left(GBest\left(t\right)-Position_{i}\left(t\right)\right) \end{array}$$

(11) $$\begin{array}{c} Position_{i}\left(t+1\right)=Position_{i}\left(t\right)+Velocity_{i}\left(t+1\right) \end{array}$$

In other words, each particle moves towards its previous best, Lbest, position and the global best, GBest, position in the swarm. This process is repeated until the termination criteria is met, that is after the given maximum number of iterations. Algorithm 4 illustrates the pseudocode for the PSO-based clustering algorithm.

- PSO Complexity

Similar to the definition of computational complexity for the *K*-means and GA algorithms presented in the previous Section 5.2 and Section 5.3.1, respectively, the computational complexity of the PSO-based clustering algorithm refers to the total number of Euclidean distance computations and the additional computations in the algorithm. More specifically, in each iteration, the Euclidean distance of each data point from each *k* cluster center, $c_{k}$, will be computed and additional computations update particles’ velocities and positions. Hence, the computational complexity can be denoted as O(nkp). Thus, for $N_{it}$ number of iterations, the computational complexity is $O(nkpN_{it})$.
**Algorithm 4:** Clustering using Particle Swarm Optimization.
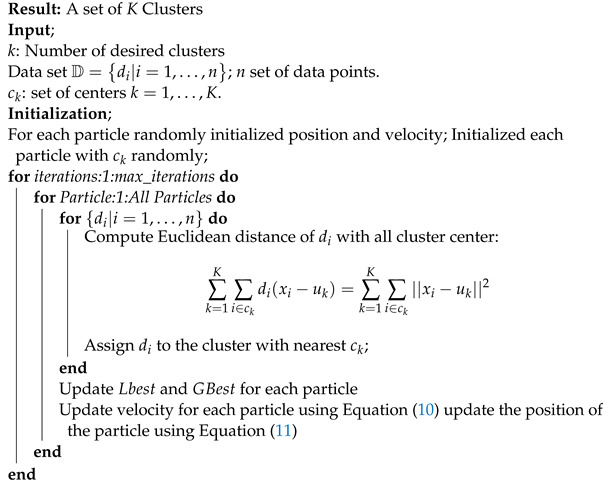


## 6. Efficient UAV 3D Placement Algorithms

As discussed in Section 4, in each iteration of the proposed heuristic algorithm, the efficient UAV 3D placement algorithm is invoked after the clustering process using one of the algorithms of Section 5. More specifically, this algorithm is used to determine efficient UAV 3D placement in each cluster, *k*.

The problem of finding an efficient UAV 3D placement is formulated with the objective to minimize its required transmit power that satisfies the users’ minimum data rate. The formulation of this problem can be found in [9,10].

Due to the intractability of the problem, efficient UAV 3D placement algorithms that invoked PSO and GA were proposed in [9,15], respectively. This paper extends the solution by invoking ABC algorithm.

Readers are referred to the discussions presented in [9,15] for detailed discussion in finding the efficient UAV 3D placement by invoking PSO and GA algorithms, respectively.

### 6.1. Problem Formulation

In this work, it is considered that UAV transmits data to $M_{out}$ outdoor and $M_{in}$ indoor users inside cluster *k* at a desired data rate, *r*, where, ($M_{out}$ + $M_{in}$) is the total number of users inside a cluster, *k* (coverage subarea) and each user has a channel bandwidth equal to *A*/($M_{out}$ + $M_{in}$), where *A* is the UAV transmission bandwidth, $L_{i}$ is the path loss between UAV and users${}_{i}$ and Np is the noise power. The total required transmit power of the UAV to satisfy the data rate, *r*, for all users inside cluster, *k* can be formulated as:

(12) $$\begin{array}{c} P_{t_{total}}=\sum_{i=1}^{\left(M_{out}+M_{in}\right)}\left(2^{\frac{R_{b}\cdot \left(M_{out}+M_{in}\right)}{A}}-1\right)NpL_{i} \end{array}$$

Thus, the problem of finding an efficient UAV 3D placement in providing wireless coverage for all users within each cluster such that the total required UAV transmit is minimized can be formulated as follows [9,10]:

(13) $$\begin{array}{c} \min_{{\left(x,y,z\right)}_{UAV}:\;\in \;k}P_{k_{Total}}=\sum_{i=1}^{M_{out}}P_{i_{out}}+\sum_{j=1}^{M_{in}}P_{j_{in}}\;\;\;\;\;\;\;\;\;\;\;\;\;\;\;\;\;\;\;\;\;\;\;\;\;\;\;\;\;\;\;\;\;\\ subject\;to\;\;\;\;\;\;\;\;\;\;\;\;\;\;\;\;\;\;\;\;\;\;\;\;\;\;\;\;\;\;\;\;\;\;\;\;\;\;\;\;\;\;\;\;\;\;\;\;\;\;\;\;\;\;\;\;\;\;\;\\ x_{min}\leq x_{UAV}\leq x_{max},\;\;\;\;\;\;\;\;\;\;\;\;\;\;\;\;\;\;\;\;\;\;\\ y_{min}\leq y_{UAV}\leq y_{max},\;\;\;\;\;\;\;\;\;\;\;\;\;\;\;\;\;\;\;\;\;\;\\ z_{min}\leq z_{UAV}\leq z_{max},\;\;\;\;\;\;\;\;\;\;\;\;\;\;\;\;\;\;\;\;\;\;\\ P_{k_{Total}}\leq P_{max}\;\;\;\;\;\;\;\;\;\;\;\;\;\;\;\;\;\;\;\;\;\;\;\;\;\;\; \end{array}$$

Here, the first three constraint equations represent the minimum and maximum allowed 3D placement for $x_{UAV}$, $y_{UAV}$ and $z_{UAV}$. In the fourth constraint equation, $P_{max}$ is the maximum allowable power, where $P_{t,max}$ is the maximum transmit power of UAV.

### 6.2. Artificial Bees Colony (ABC)

The Artificial Bees Colony (ABC) algorithm is a meta-heuristic algorithm based on the foraging behavior observed in honey bee swarms, which was introduced by Dervis Karaboga in 2005 [47].

In the ABC algorithm, the colony consists of three groups of bees, namely employed bees, onlooker bees and scout bees. Algorithm 5 shows the pesudocode of the efficient UAV 3D placement that invokes ABC algorithm. The algorithm consists of the following main steps:

1. Initialization: In the initialization phase, $N_{pop}$ random solutions are generated. In the ABC algorithm, a solution to the optimization problem is referred to as food source, $\theta_{i}$.
2. Employed bee phase: In this phase, each employed bee which has been assigned to a food source, $\theta_{i}$ searches the neighboring region to seek the best food source. The best food source is selected using greedy selection. More specifically, in this phase, the employed bee seeks a new food source, that is denoted as $\upsilon_{i}$, around the assigned source. Then, the employed bee evaluates and compares the quality of the nectar of the assigned food source, $\theta_{i}$, and the new food source, $\upsilon_{i}$. If the new food source, $\upsilon_{i}$, results in better nectar quality, then the food source, $\theta_{i}$, will be replaced by $\upsilon_{i}$; otherwise, $\theta_{i}$ remains in the population. This selection process is known as greedy selection. The nectar quality evaluation refers to the evaluation of the objective function to the problem of finding UAV 3D placement.
3. Onlooker bee phase: Then, each employed bee returns to its hive and shares the food source location with the onlooker bees that are waiting in the hive. In this phase, the quality of the nectar from all of the employed bees is evaluated. The onlooker bee selects the food source by applying the roulette wheel selection. Then, the onlooker bee searches the neighboring region of the selected food source further. The onlooker bee performs a similar selection process in the employed bee phase where the best food source is selected using greedy selection, where the better one survives in the population.
4. Scout bee phase: If a food source cannot be improved any more, the food source is abandoned or eliminated from the population. In this work, the abandonment limit parameter is defined as $0.6\times nVar\times N_{pop}$, where nVar is the dimension of the solution and refers to the 3D coordinate of (x,y,z) and $N_{pop}$ is the population size. This is carried out by replacing it with a random number. The employed bee whose food source has been abandoned becomes a scout bee and is assigned to a random new food source.
5. Termination criteria: If the termination criterion is not met, the employed bee phase, the onlooker bee phase and the scout bee phase will be repeated. In this work, the maximum number of iteration, $N_{it}$ is set as the termination criteria. The best food source will remain in the population as the best solution to the optimization problem.

**Figure 3:**
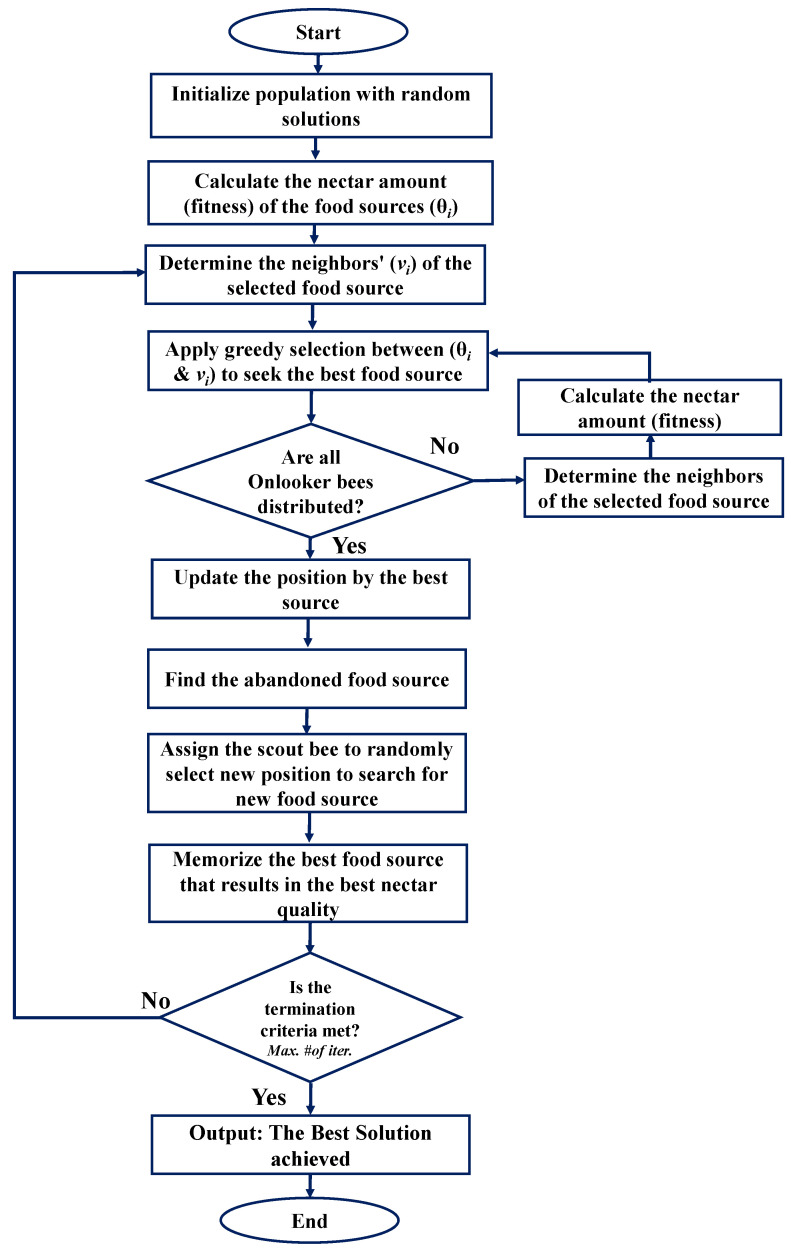
Flowchart of the Artificial Bees Colony (ABC) algorithm.

Moreover, Figure 3 presents the flowchart of the ABC algorithm.
**Algorithm 5: **Artificial Bees Colony (ABC) algorithm.**Input**: Coverage subarea S; *n* set of users location.**Initialization**: Initialize population with random solutions;**Repeat:**       Assign the employed bees to their respective food sources       Calculate the fitness of the new food source       Apply Greedy selection process       Assign the onlooker bees to the selected food sources with the best quality of nectar using roulette wheel selection       Identify the food source to be abandoned       Assign the scout bee to randomly select new area to search for new food source       Memorize the best food source that results in the best nectar quality (Best food source found so far)**Until**: The termination criteria is met.**Output:** The Best Solution achieved.

## 7. Simulation Results

This section is structured as follows. Section 7.1 presents the performance comparison of clustering algorithms in Section 5 to partition all users inside the disaster-affected area, S. Then, the performance of the proposed power-efficient algorithm is presented in Section 7.2.

### 7.1. Performance Comparison of Clustering Algorithms

In this section, we comparatively study the performance of the iterative distance-based and meta-heuristic based clustering algorithms that are presented in Section 5. The simulation results were obtained when each clustering algorithm was employed to partition outdoor and indoor users that were uniformly distributed. The user partitioning corresponds to the partitioning of the disaster-affected area, S.

**Figure 4:**
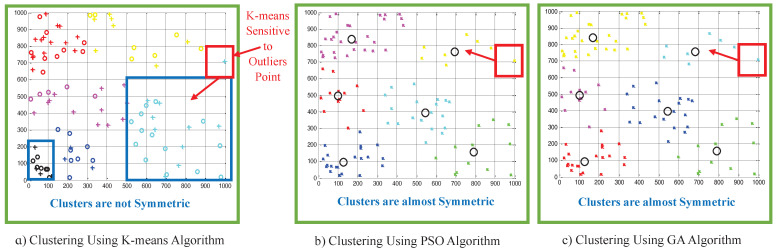
Clustering results using (**a**) *K*-means, (**b**) PSO-based and (**c**) GA-based clustering algorithms.

Figure 4 shows the clustering results using the three clustering algorithms where the users are partitioned into six clusters. The users in each cluster are indicated with the same color. It can be seen from Figure 4a that the *K*-means algorithm produces clusters that are not symmetric, as shown by the clusters marked with the blue square. This observation is consistent with the results presented in [34]. Meanwhile, the meta-heuristic based clustering algorithms, namely the PSO-based and GA-based algorithms, form symmetric clusters.

It can also be observed in Figure 4a that an outlier that is marked with a red square affects the clustering process of the neighboring data points. The outlier resulted in a heavily unbalanced cluster, as marked with blue square. In other words, this indicates that the outlier resulted in some data points being clustered incorrectly [32]. Meanwhile, the same outlier data point (marked with red square) did not affect the clustering results when PSO-based and GA-based algorithms were employed, as shown in Figure 4b,c.

Moreover, it can also be seen from Figure 4b,c that meta-heuristic algorithms lead to the same clustering results [31].

The efficiency of each clustering algorithm is evaluated in terms of its execution time. In this work, the simulations were conducted using a laptop with the following specifications: Intel core i7-4710HQ CPU with 3.1 GHz processor and 8 GB RAM in Windows 10 OS. Table 1 presents the execution time taken to partition all users that are uniformly distributed into *K* clusters using *K*-means, PSO-based and GA-based clustering algorithms. The execution time taken by each clustering algorithm reflects the computation complexity defined in Section 5.1. More specifically, the *K*-means algorithm took the shortest time to complete the clustering process compared to the meta-heuristic-based clustering algorithms. Meanwhile, the employment of the GA-based clustering algorithm resulted in inferior performance compared to that of the PSO-based clustering algorithm. More specifically, the GA-based algorithm took a longer time to partition all users into clusters.

Therefore, the performance of the proposed power-efficient algorithm of Algorithm 1 in Section 4 is evaluated by invoking *K*-means clustering and PSO-based clustering algorithms. Moreover, the GA-based clustering algorithm produces similar clustering results as that of the PSO-based clustering algorithm, but at the expense of higher complexity due to the crossover and mutation operators.

### 7.2. Performance of Power-Efficient Algorithm

This section presents the performance of the proposed power-efficient algorithm of Algorithm 1 in Section 4. Table 2 summarizes the parameters used in the simulation.

**Figure 5:**
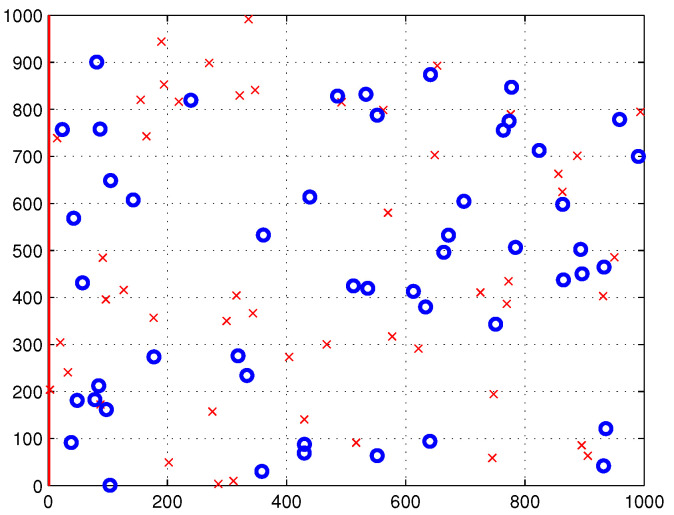
Uniformly distributed outdoor and indoor users inside S, denoted as blue circles and red crosses, respectively.

**Figure 6:**
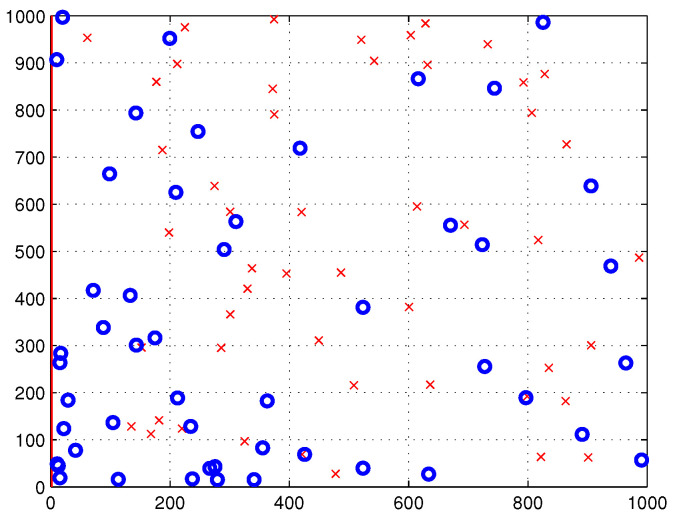
Non-uniformly distributed outdoor and indoor users inside S, denoted as blue circles and red crosses, respectively.

In this study, we consider the dimension of the coverage area, S, to be 1000m×1000m. The proposed power-efficient algorithm is evaluated in two scenarios where all users are distributed with two distributions, namely uniform and non-uniform distributions, as shown in Figure 5 and Figure 6, respectively. In this simulation, the total number of users is 100, which is composed of 50 outdoor users and 50 indoor users.

In each scenario, the power-efficient algorithm invoked the *K*-means and PSO-based clustering algorithms of Algorithms 2 and 4, respectively, to partition the users. Then, the three variants of efficient UAV 3D placement algorithms of Section 6 that invoked PSO, GA and ABC algorithms were employed. The clustering and the efficient UAV 3D placement algorithms were iteratively invoked to find the minimum number of UAVs that provide wireless coverage to the disaster-affected area, S, in such that the UAV transmit power is minimized and the users’ data rate is satisfied. In this study, the minimum users’ data rate is 1Mbps and the minimum UAV altitude $z_{min}=60\;m$. The minimum UAV altitude value during SAR operation is 60m. This value is less than 120m, the allowable height under the Drone Safety Rules [48].

More specifically, the clustering and the efficient UAV 3D placement algorithms were performed iteratively until the the UAV total transmit power constraint of Equation (4b) was met, as described in Section 4. In other words, the iteration was terminated if the UAV transmit power required to cover all outdoor and indoor users inside each cluster was less than the threshold of 1watt, which is also referred to as the maximum allowable UAV transmit power, ${P_{UAV}}_{max}$. The performance comparison of different combinations of the clustering and efficient UAV 3D placement algorithms were evaluated, in terms of the minimum total UAV required transmit power, UAV placement and algorithm execution time.

For the uniform distribution scenario, the proposed power-efficient algorithm was evaluated against the multi-UAVs deployment approach that utilized CPT as described in Section 4.

#### 7.2.1. Uniform Distribution Users Scenario

Table 3 shows the simulation results of the proposed algorithm that minimizes the number of UAVs in providing wireless coverage to all users within the disaster-affected area, S, such that the UAV required transmit power is minimized and the users’ data requirements are satisfied. The number of UAVs in Table 3, indicates the number of clusters.

It can be observed that the power-efficient algorithm that invoked the PSO-based clustering algorithm outperforms the algorithm that invoked the *K*-means clustering algorithm. More specifically, six UAVs are required to be employed in providing wireless coverage to users within the disaster-affected area, S, as evidenced by the required transmit power of each UAV is less than 1 watt. However, in the case of the proposed algorithm that invoked *K*-means clustering algorithm, it is observed that the required transmit power of $UAV_{4}$ is more than 1 watt. As explained in Section 4, the number of clusters will be increased by 1 if the threshold value of UAV required transmit power is exceeded. Thus, this indicates that more UAVs are needed when the *K*-means clustering algorithm is invoked.

**Figure 7:**
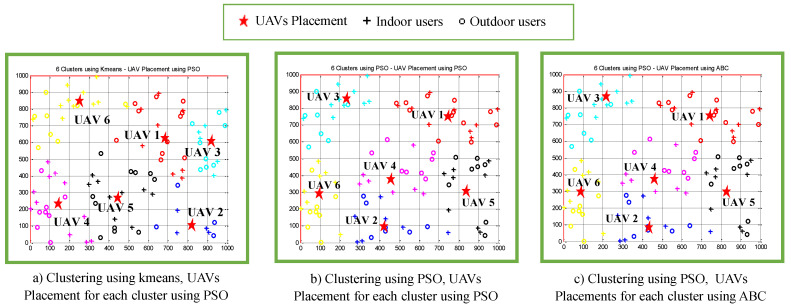
Placement of UAV1 to UAV6 when users were uniformly distributed. (**a**) Clustering using *K*-means, UAV placement using PSO. (**b**) Clustering using PSO, UAV placement using PSO. (**c**) Clustering using PSO, UAV placement using ABC.

Figure 7 shows three of the clustering results and the UAVs placements from Table 3. Figure 7a depicts the simulation results of the proposed algorithm that iteratively invoked the *K*-means clustering algorithm and the PSO-based efficient UAV 3D placement algorithms. Meanwhile, the simulation results of the proposed algorithm that iteratively invoked the PSO-based clustering algorithm and two of the variants of efficient UAV 3D placement algorithms, namely PSO-based and ABC-based efficient UAV 3D placement algorithms, are depicted in Figure 7b,c, respectively. In this case, the outdoor and indoor users are distributed uniformly, which are denoted by the circle and cross symbols, respectively. These figures illustrate that the users are partitioned into six clusters, and one UAV is assigned to each cluster, where its 2D position is denoted by star. The coordinate of the 3D placement of each UAV in Figure 7a–c is presented in Table 3. It can be observed from Table 3 that the total UAV required transmit power is at a minimum when the UAV altitude is close to the minimum UAV altitude of 60 m. This is because there is a trade-off between the UAV altitude, the probability of LoS and the coverage performance in terms of coverage radius [8]. For lower UAV altitudes, the probability of LoS between transmitter and receiver decreases due to the shadowing impact. Thus, it results in a decrease in the total UAV required transmit power of each UAV.

Figure 7a,b illustrate the comparison performance of clustering results between *K*-means and PSO clustering algorithms. From these figures, it can be clearly seen that the *K*-means algorithm produces clusters that are not symmetric, as discussed in Section 5. Meanwhile, Figure 7b,c illustrate the comparison performance of the proposed power-efficient algorithms that invoked PSO and ABC algorithms, respectively. It can be seen that both combinations produce similar performances in terms of clustering results and the UAV placements.

**Figure 8:**
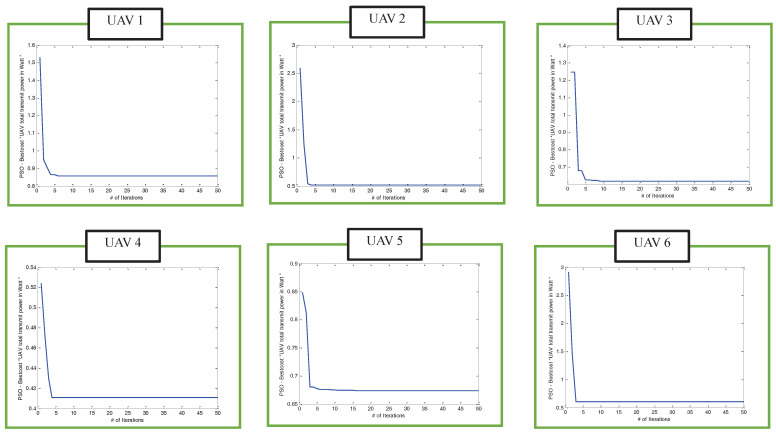
The convergence speeds of the power-efficient algorithm that iteratively invoked PSO-based clustering and PSO-based efficient algorithms for UAV1 to UAV6 when users were uniformly distributed.

Meanwhile, the convergence speed of the proposed algorithm that iteratively invoked PSO-based clustering algorithm and PSO-based efficient UAV 3D placement for the six UAVs is shown in Figure 8. It can be observed from this figure that the proposed algorithms converge to the total UAV transmit power smaller than the threshold value of 1 watt within a few iterations.

**Figure 9:**
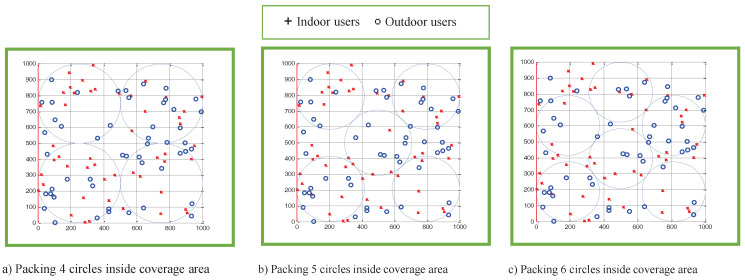
Optimal packing of (**a**) 4 circles, (**b**) 5 circles and (**c**) 6 circles using CPT inside a square region.

Figure 9 illustrates the optimal results of packing four, five and six equal circles inside the coverage area, S, using the CPT-based benchmarker algorithm. The problem of packing $n_{c}$ identical and non-overlapped circles inside a unit square for $2\leq n_{c}\leq 22$ was studied in [9]. The problem is formulated with the objective to maximize the radius $r_{d}$ of the packed circles such that the coverage density, $d_{n}$, is maximized. The coverage density, $d_{n}$, for packing $n_{c}$ identical circles was determined using Equation (7). It was observed that the maximum coverage density of 78.5% was achieved when $n_{c}$ = 4, 9 and 16 [9]. It was also observed that the large number of UAVs (indicated by $n_{c}$) did not necessarily result in the maximum coverage density [9].

Packing four, five and six circles into the coverage area, S, that has a square shape resulted in coverage density of 78.5%, 67.4% and 66.4%, respectively. Thus, the maximum achievable coverage density of packing $n_{c}$ equal circles in the coverage region is 78.5%, when $n_{c}=4$.

Readers who are specifically interested in the most efficient algorithm in finding the minimum number of UAVs that maximizes wireless coverage using CPT to serve users in large coverage areas with three different shapes, namely square, rectangle and circular regions, are referred to [9] for further information. More specifically, discussions on the problem of packing $n_{c}$ identical circles using CPT inside a unit square and three different shapes of coverage area are presented in [9].

The performance of the proposed power-efficient algorithm that invoked a PSO-based clustering algorithm is evaluated against the benchmarker algorithm with a CPT-based approach. The condition of the total required UAV transmit power to be smaller than the threshold value of 1 watt is also considered in the CPT-based benchmarker algorithm. The results in Table 4 reveal that all users were partitioned into five clusters when the CPT-based benchmarker algorithm was invoked. Therefore, the multi-UAVs deployment with the CPT approach resulted in a smaller number of UAVs being deployed in providing wireless coverage to all users, but at the expense of the coverage density of 67.4% in comparison with the 100% coverage density using the proposed power-efficient algorithm.

#### 7.2.2. Non-Uniform Distribution Users Scenario

For the case of users that are non-uniformly distributed, similar observations were obtained from Table 5. More specifically, the power-efficient algorithm that invoked a PSO-based clustering algorithm is superior in comparison to the algorithm that invoked a *K*-means clustering algorithm, as presented in Table 5. This is evident as the required transmit power for $UAV_{3}$ is more than the threshold value of 1watt when the *K*-means clustering algorithm was used to partition the users. Thus, this indicates more than six UAVs are required to be deployed in providing wireless coverage for all users within the coverage area in comparison to the algorithm that invoked the PSO-based clustering algorithm which requires six UAVs.

**Figure 10:**
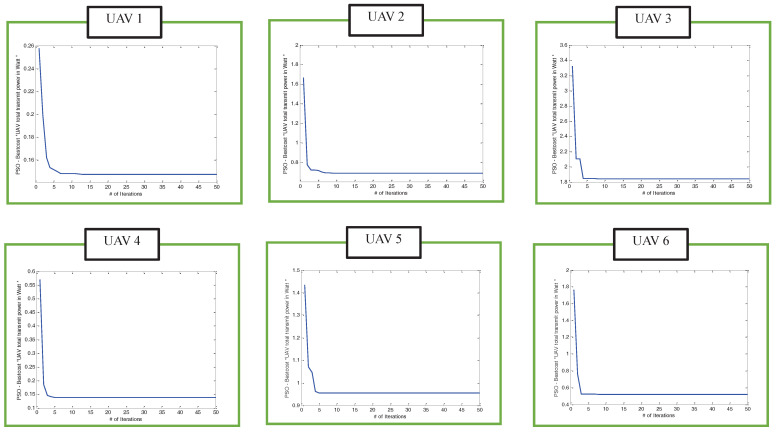
The convergence speeds of the power-efficient algorithm that iteratively invoked PSO-based clustering and PSO-based efficient algorithms for UAV1 to UAV6 when users were non-uniformly distributed.

Moreover, Figure 10 shows the convergence speed of the proposed power-efficient algorithm that iteratively invoked the PSO-based clustering algorithm and PSO-based efficient UAV 3D placement for the case of non-uniformly distributed users. A similar observation was obtained from this figure where the simulation results converge to the total UAV transmit power smaller than the threshold value of 1 watt within a few iterations for each UAV that serves each cluster of users.

**Figure 11:**
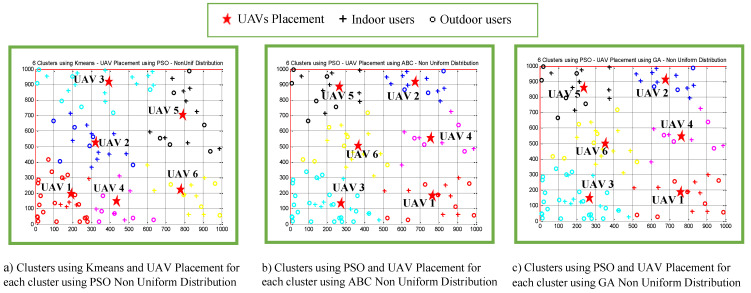
(**a**–**c**) Placement of UAV1 to UAV6 when users were non-uniformly distributed.

Figure 11 shows three of the clustering results and the UAV placements from Table 5. A similar observation was obtained from comparison of Figure 11a,b, where it can be clearly seen that the *K*-means algorithm produces clusters that are not symmetric, as discussed in Section 5. It can be seen from Figure 11b,c that the combination of the PSO-based clustering algorithm and the two variants of efficient UAV 3D placement algorithms, namely ABC-based and GA-based algorithms, produced similar performances in terms of clustering results and the UAV placements.

The corresponding computational complexity performances for the proposed algorithm in relation to Table 3 and Table 5 is shown in Table 6. In both scenarios, the execution time is used to represent the power-efficient algorithm computational complexity in finding the minimum number of UAVs to provide wireless coverage to outdoor and indoor users that are distributed uniformly and non-uniformly. It can be observed in Table 6 that the power-efficient algorithm took the shortest execution time to find the minimum number of UAVs by iteratively invoking the PSO-based clustering algorithm and the efficient UAV 3D placement algorithm based on PSO, rather than employing the other two variants of efficient UAV 3D placement algorithms.

Although the employment of the *K*-means clustering algorithm resulted in a shorter execution time when the PSO-based efficient UAV 3D placement algorithm was invoked, the resultant number of UAVs that were required to be deployed was inferior to that when the PSO-based clustering algorithm was invoked, as discussed in Section 7.1.

Therefore, it was found to be beneficial to employ PSO-based clustering and a PSO-based efficient UAV 3D placement algorithm for the case of users that were distributed uniformly and non-uniformly.

## 8. Conclusions

In this work, a power-efficient algorithm was proposed for minimization of the number of UAVs to be deployed as aerial base stations to serve outdoor and indoor users simultaneously, which led to maximum users’ coverage using the minimum number of UAVs, such that the required UAV transmit power was minimized.

In this study, three variants of clustering algorithms based on the *K*-means algorithm, PSO and GA were employed. It was observed that meta-heuristic clustering algorithms, namely GA-based and PSO-based, were superior in comparison to the *K*-means algorithm that resulted in a smaller number of user clusters.

It was observed that the proposed algorithm that iteratively invoked the PSO-based clustering algorithm and the PSO-based efficient UAV 3D placement algorithm has the lowest computational complexity compared to the proposed algorithm that invoked the efficient UAV 3D placement algorithm based on GA and ABC. The computational complexity performance was manifested in terms of the algorithm execution time. More specifically, for the uniformly distributed users scenario, the proposed algorithm that iteratively invoked the PSO-based clustering algorithm and the PSO-based efficient UAV 3D placement algorithm took about 0.0971 s to find the minimum number of UAVs which were positioned at their efficient UAV 3D placements.

The proposed algorithm was evaluated against a benchmarker algorithm that utilized CPT and efficient UAV 3D placement for a multi-UAV deployment scheme. It was observed that the benchmarker algorithm required a lower number of UAVs to be deployed as aerial base stations, but at the expense of the coverage density of 67.4% in comparison with 100% coverage density using the proposed power-efficient algorithm. The employment of the proposed algorithm resulted in 100% coverage density, which was manifested by its achievement to serve all users within the disaster-affected area, S.

## Figures and Tables

**Table 1:** Execution time for clustering algorithms to partition uniformly distributed users.

		Execution Time in Seconds	
Clustering Algorithm	Algorithm Complexity	Uniform Distribution	Non-Uniform Distribution
*K*-means	O(nkL)	0.042	0.0685
PSO	O(nkpL)	0.142	0.3824
GA	O(L·nklog(k))	3.1492	3.2331

**Table 2:** Simulation and system parameters.

Simulation Parameters			System and Algorithms Parameters		
Subarea (S) dimensions	($x_{max},y_{max}$)	(1000 m, 1000 m)	Number of Decision Variables	nVar	3
UAV altitude	$Z_{min}$	60 m	# of Individuals (GA selectionphase)	*k*-individuals	4
Number of outdoor users	$M_{out}$	50	ABC Abandonment Limit Parameter	$L_{limit}$	$0.6*nVar*N_{pop}$
Number of indoor users	$M_{in}$	50	ABC—Number of Onlooker Bees	nOnlooker	50
Carrier frequency	$f_{c}$	2 GHz	ABC, PSO, GA Max # of iterations	$N_{it}$	50
Noise power	Np	−100 dBm	ABC, PSO and GA Population size	$N_{pop}$	100
Data Rate	*r*	1 Mbps	Indoor Environment parameter	$a_{1},a_{2},a_{3},a_{4}$	31.4, 15, 14, 0.5
Total Bandwidth	B	50 MHz	Outdoor Environment parameter	α,β	9.6, 0.28
Max. UAV transmit power	$P_{t_{UAV_{max}}}$	1 watt	Outdoor Environment parameter	$\eta_{LSO},\eta_{NLSO}$	1, 20

**Table 3:** Simulation results of the power-efficient algorithm for uniform distribution scenario.

Cluster	Clustering	PSO Alg. UAV	ABC Alg. UAV	GA Alg. UAV
UAV#	Algorithm	Placement + Power	Placement + Power	Placement + Power
UAV${}_{1}$	*K*-means	[685.8893 623.2211 60]:0.8546 watt	[689.1949 622.4364 60.292]:0.8574 watt	[685.7911 623.7972 60.000]:0.8547 watt
	PSO	[744.4978 749.1702 60]:0.8574 watt	[742.6962 752.2831 60.189]:0.8584 watt	[744.5963 748.8969 60.012]:0.8574 watt
UAV${}_{2}$	*K*-means	[820.3453 103.3693 60]:0.063 watt	[827.827 98.48832 60.9426]:0.064 watt	[837.6544 118.0251 62.7099]:0.068 watt
	PSO	[421.8992 94.72741 60]:0.517 watt	[434.3266 83.9485 60.3107]:0.522 watt	[419.5079 100.2885 60.4114]:0.517 watt
UAV${}_{3}$	*K*-means	[921.5019 608.1418 60]:0.2544 watt	[923.3534 608.0932 60.042]:0.2546 watt	[929.4961 610.5776 60.544]:0.2563 watt
	PSO	[234.0778 854.9350 60]:0.6173 watt	[216.8301 866.1314 60.298]:0.6356 watt	[230.4671 846.5856 62.311]:0.6400 watt
UAV${}_{4}$	*K*-means	[144.2928 233.3356 60]:1.6854 watt	[145.3373 236.797 60.7461]:1.6909 watt	[143.4650 234.4760 60.079]:1.6855 watt
	PSO	[457.0585 375.8655 60]:0.4109 watt	[462.1730 371.6102 61.888]:0.4161 watt	[456.7381 375.6460 60.023]:0.4110 watt
UAV${}_{5}$	*K*-means	[445.4709 268.8294 60]:0.6294 watt	[440.3215 261.6336 60.408]:0.6346 watt	[443.7819 271.0478 60.351]:0.6315 watt
	PSO	[838.0584 306.6074 60]:0.6739 watt	[829.1685 295.4001 62.404]:0.6847 watt	[841.5975 311.6093 62.432]:0.6817 watt
UAV${}_{6}$	*K*-means	[252.3852 845.8628 60]:0.9052 watt	[253.8206 847.070 60.357]:0.9081 watt	[253.4335 846.8824 61.519]:0.9171 watt
	PSO	[96.30438 291.2943 60]:0.6069 watt	[87.6339 297.0693 60.089]:0.6100 watt	[100.2821 288.2098 60.146]:0.6082 watt

**Table 4:** Simulation results of the CPT-based benchmarker algorithm for uniform distribution scenario.

Packed	Circle	Coverage	PSO Alg. UAV	ABC Alg. UAV	GA Alg. UAV
Circle, UAV#	Radius	Density	Placement + Power	Placement + Power	Placement + Power
UAV${}_{1}$	207.11	67.37%	[167.1695 219.9231 60]:0.6002 watt	[168.7157 217.7514 60.0000]:0.6002 watt	[172.7874 228.5162 60.0258]:0.6021 watt
UAV${}_{2}$			[783.0499 220.4436 60]:0.4694 watt	[789.3028 198.8249 60.33249]:0.4756 watt	[697.2269 204.0371 62.7439]:0.5552 watt
UAV${}_{3}$			[412.758 389.7928 60]:0.35099 watt	[423.4603 395.749 60.25453]:0.3532 watt	[410.0991 392.8970 60.0000]:0.3512 watt
UAV${}_{4}$			[230.5809 846.9975 60]:0.4593 watt	[239.4186 856.8646 60.4238]:0.4715 watt	[220.0381 868.8871 60.2618]:0.4765 watt
UAV${}_{5}$			[810.9745 737.4375 60]:0.3658 watt	[821.9865 733.8699 60.6021]:0.3697 watt	[812.6761 732.1948 60.0000]:0.3664 watt

**Table 5:** Simulation results of the power-efficient algorithm for non-uniform distribution scenario.

Cluster	Clustering	PSO Alg. UAV	ABC Alg. UAV	GA Alg. UAV
UAV#	Algorithm	Placement + Power	Placement + Power	Placement + Power
UAV${}_{1}$	*K*-means	[193.0913 195.5615 60]:0.1471 watt	[195.6734 202.5016 60.058]:0.1481 watt	[189.7199 197.735 60.000]:0.1473 watt
	PSO	[765.2653 178.5259 60]:0.5373 watt	[765.0626 182.1474 60.698]:0.5402 watt	[758.8278 185.712 65.197]:0.5587 watt
UAV${}_{2}$	*K*-means	[322.8127 523.4081 60]:0.6871 watt	[327.4116 523.0595 60.189]:0.6887 watt	[325.1751 525.468 60.000]:0.6874 watt
	PSO	[681.2329 904.7334 60]:0.4773 watt	[673.7837 916.3648 60.149]:0.4822 watt	[679.2162 909.421 61.797]:0.4865 watt
UAV${}_{3}$	*K*-means	[394.1480 917.7155 60]:1.8389 watt	[394.9405 912.3090 60.313]:1.8425 watt	[407.901 871.4571 62.349]:1.9651 watt
	PSO	[268.8858 147.3919 60]:0.6180 watt	[275.8520 132.4072 60.913]:0.6284 watt	[269.2245 147.445 60.000]:0.6180 watt
UAV${}_{4}$	*K*-means	[436.3670 146.9905 60]:0.1385 watt	[446.9090 135.1591 60.256]:0.1406 watt	[438.2020 147.3331 60.00]:0.1385 watt
	PSO	[761.7588 546.8866 60]:0.3653 watt	[756.2841 553.2898 61.699]:0.3694 watt	[763.2174 546.104 69.191]:0.3847 watt
UAV${}_{5}$	*K*-means	[791.0486 704.5362 60]:0.9549 watt	[800.0364 708.7597 61.913]:0.9649 watt	[788.6125 697.887 61.253]:0.9609 watt
	PSO	[256.3875 875.1078 60]:0.4577 watt	[265.5788 884.0287 60.249]:0.4616 watt	[237.6659 858.223 71.108]:0.5153 watt
UAV${}_{6}$	*K*-means	[780.0924 222.0847 60]:0.5207 watt	[766.9253 207.0314 61.019]:0.5288 watt	[793.1164 222.281 60.462]:0.5257 watt
	PSO	[353.6876 497.1714 60]:0.6524 watt	[367.5214 504.7673 60.095]:0.6634 watt	[352.8407 499.851 60.273]:0.6544 watt

**Table 6:** Execution time for the power-efficient algorithm.

Clustering Algorithm	Users Distribution	Execution Time in Seconds		
		Efficient UAV 3D Placement Algorithm		
		PSO	GA	ABC
PSO	Uniform	0.0971 s	1.6540 s	7.9114 s
*K*-means	Uniform	0.0900 s	1.5781 s	7.0895 s
PSO	Non-Uniform	0.0925 s	1.4645 s	7.3660 s
*K*-means	Non-Uniform	0.0683 s	1.1532 s	5.6793 s
